# Review on Stress Tolerance in *Campylobacter jejuni*

**DOI:** 10.3389/fcimb.2020.596570

**Published:** 2021-02-04

**Authors:** Se-Hun Kim, Ramachandran Chelliah, Sudha Rani Ramakrishnan, Ayyappasamy Sudalaiyadum Perumal, Woo-Suk Bang, Momna Rubab, Eric Banan-Mwine Daliri, Kaliyan Barathikannan, Fazle Elahi, Eunji Park, Hyeon Yeong Jo, Su-Bin Hwang, Deog Hwan Oh

**Affiliations:** ^1^Food Microbiology Division, Food Safety Evaluation Department, National Institute of Food and Drug Safety Evaluation, Cheongju, South Korea; ^2^College of Agriculture and Life Sciences, Kangwon National University, Chuncheon, South Korea; ^3^School of Food Science, Department of Food Science and Biotechnology, College of Agriculture and Life Sciences, Kyungpook National University, Daegu, South Korea; ^4^Department of Bioresource Engineering, McGill University, Montreal, QC, Canada; ^5^Department of Food and Nutrition, College of Human Ecology and Kinesiology, Yeungnam University, Gyeongsan, South Korea

**Keywords:** *Campylobacter*, stress, resistance mechanisms, stress adaptation, enteric bacteria

## Abstract

*Campylobacter* spp. are the leading global cause of bacterial colon infections in humans. Enteropathogens are subjected to several stress conditions in the host colon, food complexes, and the environment. Species of the genus *Campylobacter*, in collective interactions with certain enteropathogens, can manage and survive such stress conditions. The stress-adaptation mechanisms of *Campylobacter* spp. diverge from other enteropathogenic bacteria, such as *Escherichia coli*, *Salmonella enterica* serovar Typhi, *S. enterica* ser. Paratyphi, *S. enterica* ser. Typhimurium, and species of the genera *Klebsiella* and *Shigella*. This review summarizes the different mechanisms of various stress-adaptive factors on the basis of species diversity in *Campylobacter*, including their response to various stress conditions that enhance their ability to survive on different types of food and in adverse environmental conditions. Understanding how these stress adaptation mechanisms in *Campylobacter*, and other enteric bacteria, are used to overcome various challenging environments facilitates the fight against resistance mechanisms in *Campylobacter* spp., and aids the development of novel therapeutics to control *Campylobacter* in both veterinary and human populations.

**Graphical Abstract:**
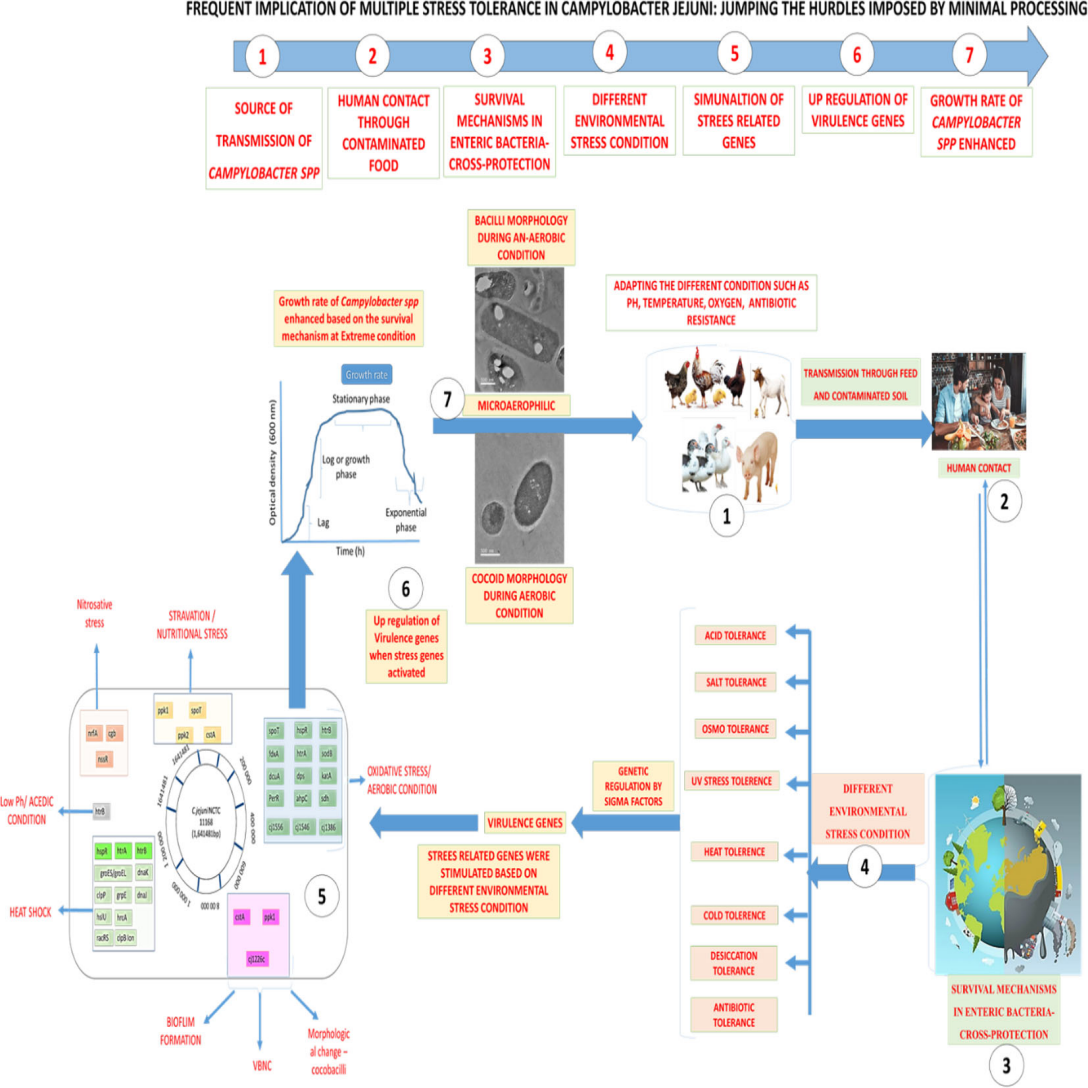
Overall flow chart on Stress Tolerance in Campylobacter jejuni at different environment condition 1. Source of transmission of Campylobacter species, 2. Human contact and throughout contaminated food, 3. Survival mechanism in entric-bacterial pathogens, 4. Differental environmental adverse condition, 5. Stimulation of stress related genes involved in sustainability, 6. Up regulation (over expression) of virulence genes, 7. Growth rate and survival of Campylobacter species enhanced.

## Introduction

**Figure 1:**
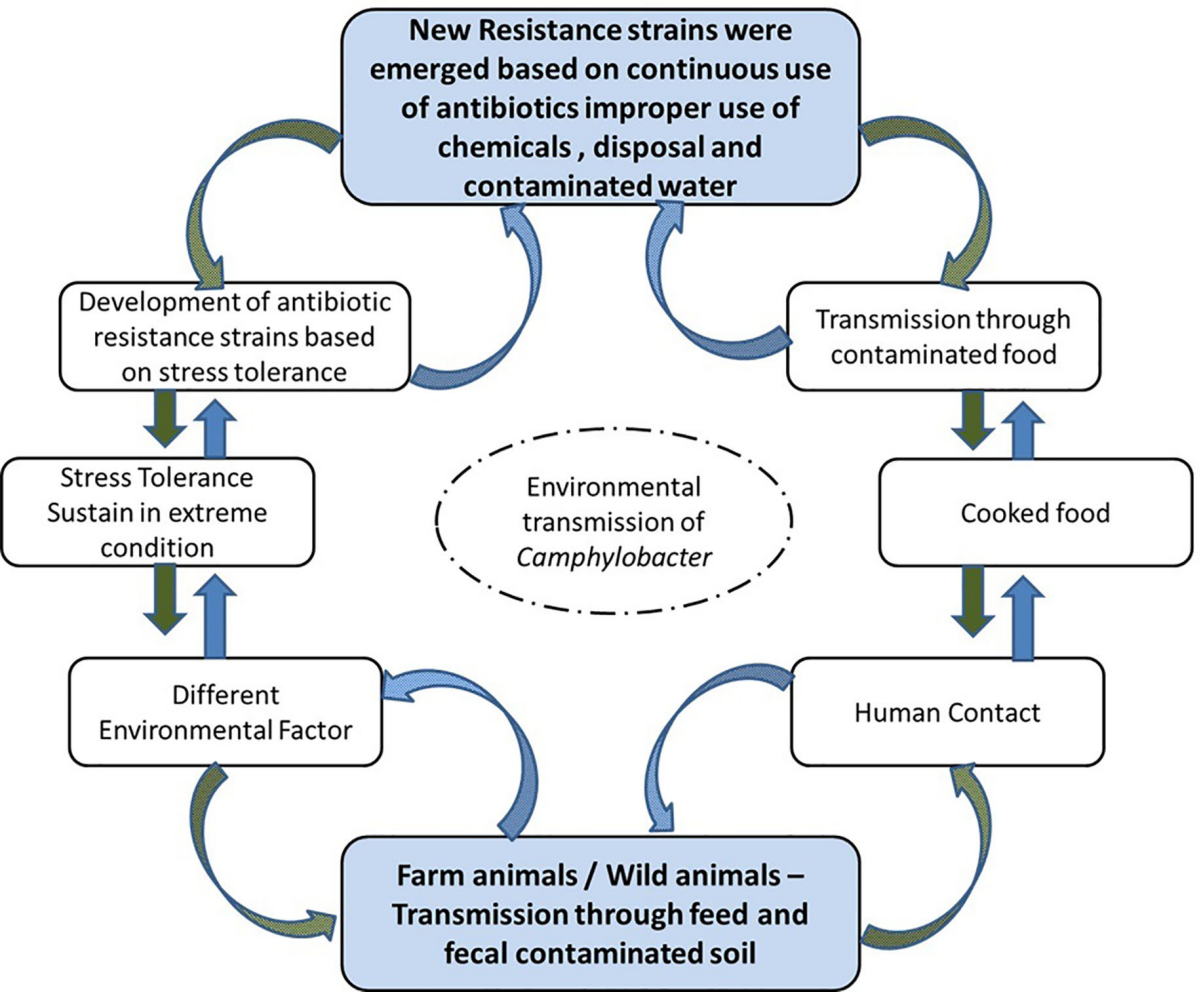
Modes of transmission for *C. jejuni*.

*Campylobacter* are a Gram-negative, slender, microaerophilic bacteria with a spiral or curved shape (0.2–0.8 mm × 0.5–5 mm). All species of the genus *Campylobacter*, with the exception of *Campylobacter gracilis* (nonmotile) and *Campylobacter showae* (peritrichous flagella), have a single, polar, unsheathed flagellum on one or both sides of the cell. Infection with *Campylobacter* in humans predominantly occurs through handling and ingestion of *Campylobacter*-contaminated raw or undercooked meat, raw milk, tap water, chicken salad, and various chicken-containing dishes (Zhao et al., 2003; Jang et al., 2007; Pedersen et al., 2018; Ovesen et al., 2019; The et al., 2019) as illustrated in **Figure 1**. Most *Campylobacter* infections involve a mild and self-limiting gastroenteritis, with one to three days of fever and vomiting, followed by abdominal pain with watery or bloody diarrhea for three to seven days (Negretti et al., 2019).

The species *Campylobacter jejuni* is a zoonotic pathogen that frequently causes acute gastrointestinal infections in humans when undercooked or raw meat or other products are consumed. Fever, vomiting, abdominal pain, and diarrhea are the prevalent symptoms of campylobacteriosis (Altekruse et al., 1999; Gaynor et al., 2005). In some cases, *C. jejuni* is associated with bacteremia and several post-infectious complications in humans, including immunoreactions and chronic and life-threatening paralysis, such as Guillain–Barré syndrome (GBS) and Miller Fisher syndrome (MFS) (Humphrey et al., 2007; EFSA, 2011).

*C. jejuni* possesses novel regulatory factors for stress resistance that enable the organism to cause foodborne infections (CDC, 2013). In most pathogens, sigma factor RpoS plays a key role in the stress-resistance mechanisms, but this factor has been reported to be absent in *C. jejuni* (Allen et al., 2018; Cain et al., 2019). *Campylobacter* is a foodborne pathogen with high incidence with norovirus, enteropathogenic *Escherichia coli*, and *Salmonella* in South Korea (Kim et al., 2017; Wang et al., 2020).

The prevalence of thermophilic *Campylobacter* for poultry is *C. jejuni* (6.3%), *C. upsaliensis* (5.9%), and *C. coli* (0.7%). Globaly 20.9% *C. jejuni* are resistant to (fluoro)quinolones. Poultry become colonized shortly after birth; commercial broilers are often particularly colonized with *C. jejuni* (EFSA, 2010), the major transmission of *C. jejuni* occurs in small intestinal crypts of poultry within 24 hours (Coward et al., 2008). *Campylobacter* can reach densities as high as 1 × 10^8^ colony-forming units (CFU/g) in the infected bird’s intestinal mucosa are asymptomatic (Meade et al., 2009). *C. jejuni* spreads to a small intestine of the gastrointestinal tract, sometimes asymptomatically, after human consumption. The onset of illness is affected by the immune status of the host and the virulence of the *Campylobacter* strain.

The pathogenesis of *C. jejuni* foodborne illness involves adhesions, gut-wall invasion, colonization, and ultimately the release of toxins (Bang et al., 2003; Bolton, 2015; Pedersen et al., 2018). Motility of this pathogen is a key factor influencing colonization and survival in the acidic gut environment (Guerry, 2007; Mehat et al., 2018; Negretti et al., 2019). Flagella-oriented genes such as *flaA* and *flaB*, and *fliF*, *fliM*, and *fliY* are associated with motility-engaged *C. jejuni* (Nachamkin et al., 1993; Wassenaar et al., 1993; Carrillo et al., 2004; Sommerlad and Hendrixson, 2007; Lertsethtakarn et al., 2011). Some Gram-negative bacteria secrete a cytolethal distending toxin (CDT) heat-labile exotoxin and able to induce the distension and death of eukaryotic cells, and this has been demonstrated in *Campylobacter* (Bolton, 2015; Scuron et al., 2016; Pedersen et al., 2018; El-Tawab et al., 2019), which synthesizes this toxin using the genes *cdtA*, *cdtB*, and *cdtC* (Linton et al., 2000; Asakura et al., 2007; Wieczorek et al., 2018). Motility, adherence, invasion, and toxin production are required for cell lysis (Bang et al., 2003). The flagellar guidance of the motility scheme and a chemosensory mechanism that activates flagellar motion result in transmission from the environment to the small bowel (O’Sullivan et al., 2018). *Campylobacter* has extraordinary motility, particularly in gelatinous or viscous material, as indicated by its single or bipolar flagella and helical filamentous structures. The polar flagellum delivers driving torque and rotating metabolic signals, while corkscrew rotation is possible due to the helical form (Ferrero and Lee, 1988). Mucins and glycoproteins, the predominant components of mucus, are the primary chemical attractants during propagation (Hugdahl et al., 1988; Wadhams and Armitage, 2004; Wuichet et al., 2007; Ellström et al., 2016). Iron acquisition also plays a key role in infection with *Campylobacter* (Baillon et al., 1999; Bang et al., 2003; Eucker and Konkel, 2012).

The purpose of this review was to examine the mechanisms that enable *Campylobacter* spp. to survive outside the host environment and remain a threat to public health. A summary of specific survival-based resistance genes is also provided. This information helps identify future pathways to eradicate and control outbreaks of *C. jejuni*.

## General Survival Mechanisms in Enteric Bacteria: Micro-Organism Cross-Protection

An extraordinary characteristic of bacteria is their ability to tolerate extreme environmental conditions or stressors. They not only tolerate ecological stress, but also adapt to different situations such as pressure, temperature, acidity, ultraviolet radiation, dehydration, susceptibility to antibiotics, and salinity. These characteristics raise some questions. Why and how do microbes in these environments survive? What biological mechanisms can we observe from these unique lifestyles? How can we use our understanding or resources to address these conditions, such as pH or temperature, to enhance or slow the growth of microbes?

**Figure 2:**
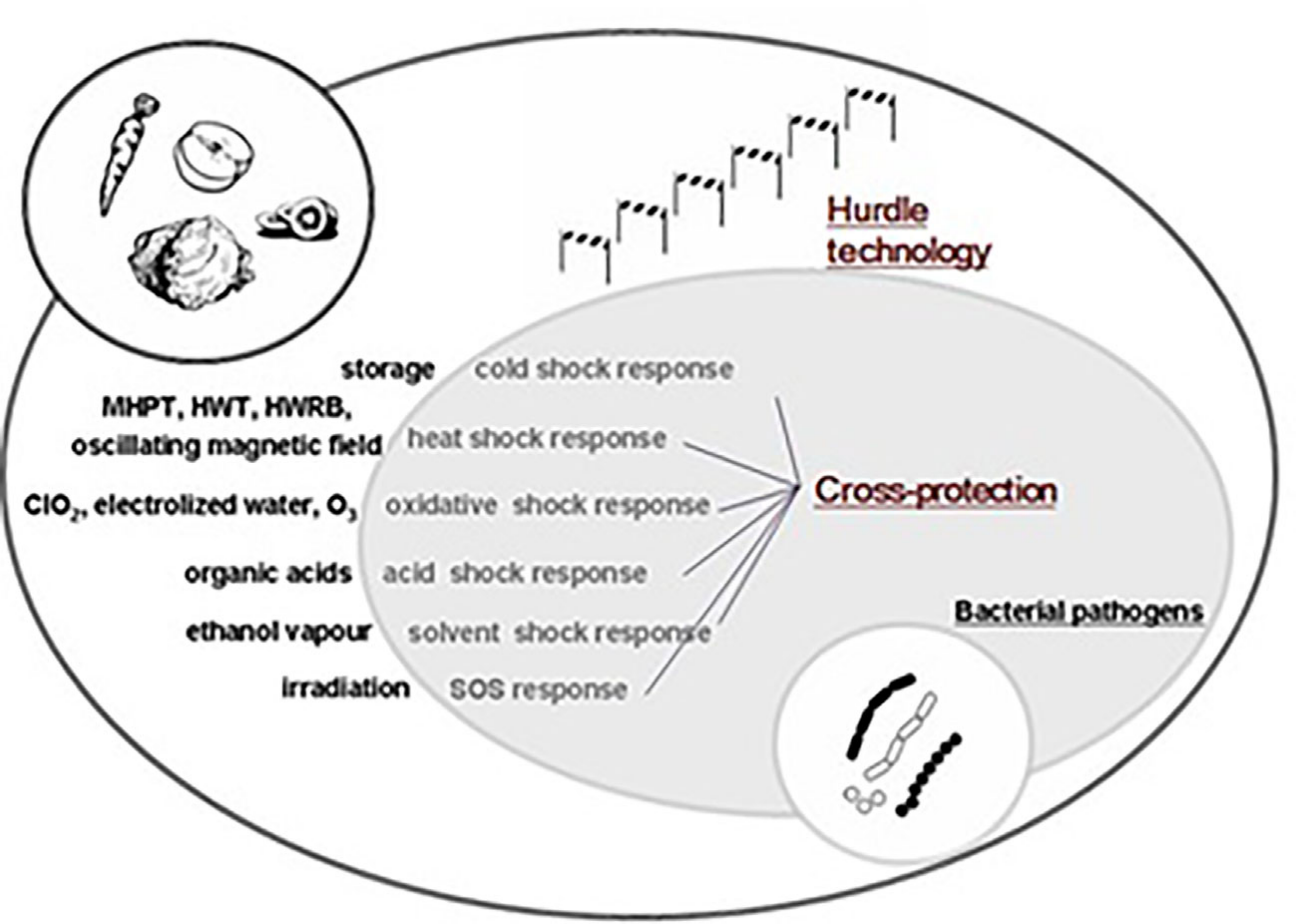
Influencing factors for foodborne pathogens.

Micro-organisms commonly face stress or shock during food processing (Ma et al., 2014). Microbes can survive in stressful or adverse environments, and can then tolerate other comparable stressors following the initial stress conditions (Isohanni et al., 2013). Cross-protection capabilities have been identified in *Salmonella* spp., *E. coli*, *Listeria monocytogenes*, and *Cronobacter sakazakii* (Kim et al., 2012; Spector and Kenyon, 2012; Lapierre et al., 2016; Wieczorek et al., 2018). For *C. jejuni*, a higher resistance to stress was observed following exposure to previous stressful environments. *C. jejuni* displayed tolerance or resistance to acid due to acquaintance with acid-aerobic, acidic, and nutrition-deprived stress (Oh et al., 2017), as well as showing oxidative stress cross-protection resulting from acid disturbance (Xu et al., 2019). However, Isohanni and Lyhs (Isohanni et al., 2013) stated that after exposure to heat and cold, *C. jejuni* did not have any cross-protection capacity, as shown in **Figure 2**.

Evidence indicates that antimicrobial agents are not used or are used incorrectly for the production of resistance *Campylobacter* spp. (Pedersen et al., 2018). Patients generally recover from campylobacteriosis without antimicrobial therapy, with treatment based on electrolyte substitution and rehydration. Severe cases can be managed with antibiotics such as tetracycline and macrolides (fluoro) or quinolones, but increases in antibiotic resistance in *C. jejuni* and *C. coli* has jeopardized the effectiveness of these therapeutics (Alfredson and Korolik, 2007; Bolinger et al., 2018).

Early in the food supply chain, *C. jejuni* is exposed to oxidative and desiccation stresses. *Campylobacter* are especially susceptible to the former as a processing technique (Humphrey et al., 1995), and in slaughter facilities, survival of *Campylobacter* in pig, and chicken meat decreases significantly by air-chill-drying the carcass surface (Oosterom et al., 1983). No comparable technique is used during the processing of poultry, and the chilling method initiates the formation of a moist surface that helps bacteria thrive (Butzler and Oosterom, 1991). Due to incomplete oxygen reduction, aerobic respiration generates reactive oxygen species (ROS), including superoxide anions (O_2–2_) and hydrogen peroxide (H_2_O_2_), which can lead to the formation of the extremely poisonous hydroxyl radical (HO). *Campylobacter* in the chicken or human body can also be subjected to H_2_O_2_ or O_2_ by the immune system to kill the microbes (Melo et al., 2019). The range of enzymes such as catalase, glutathione, cytochrome, peroxidases, peroxiredoxin alkyl hydroperoxide reductase, superoxide dismutase, and other peroxiredoxins are activated in Campylobacter exposed to ROS and these then facilitate long-term aerobic adaptation of the bacteria (Storz and Imlay, 1999) to facilitate long-term aerobic adaptation (Jones et al., 1993; Klancnik et al., 2009). *C. jejuni* has one catalase, KatA, which supports this process when the cytoplasmic level of H_2_O_2_ is high (Bingham-Ramos and Hendrixson, 2008; Melo et al., 2019).

Thermophilic species of *Campylobacter*, like *C. jejuni*, multiply at 37 to 42°CC and are unable to grow at temperatures below 30°CC (optimal growth is at 41.5°CC). At different stages of food processing, *Campylobacter* are exposed to chilled (0–4°CC) and elevated (>37–42°CC) temperatures. Evidence has shown that the response of *Campylobacter* to colder conditions (Hazeleger et al., 1998; Park, 2002) results in the slowest growth at 30°C. Low temperatures, freezing, and thawing impact different kinds of wastewater (particularly those concerning public health) and their long-term survival of enteric microbes (Zhang et al., 2009; Dasti et al., 2010; Hazeleger et al., 1998). Differences in at least 15 distinct genes were recorded between bacterial-cell and human-body temperatures of 37–42°CC, which is within the range of chicken-body temperatures. Around 48.1% of *C. jejuni* isolates showed resistance to tetracycline, and subsequent resistance to nalidixic acid (5.5%), ciprofloxacin (5.5%), azithromycin (1.78%), and erythromycin (1.78%) (Narvaez-Bravo et al., 2017). Dasti et al. (2010) reported ciprofloxacin resistance ranging from 4 μg to 32 μg/ml for the minimal inhibitory concentration. Most ciprofloxacin-resistant strains were divided into three major clonal complexes (ST-21, 48, and 353) by multilocus assessment, whereas both antibiotic-resistant strains were uniquely grouped into ST-45.

## Other General Survival Mechanisms

**Figure 3:**
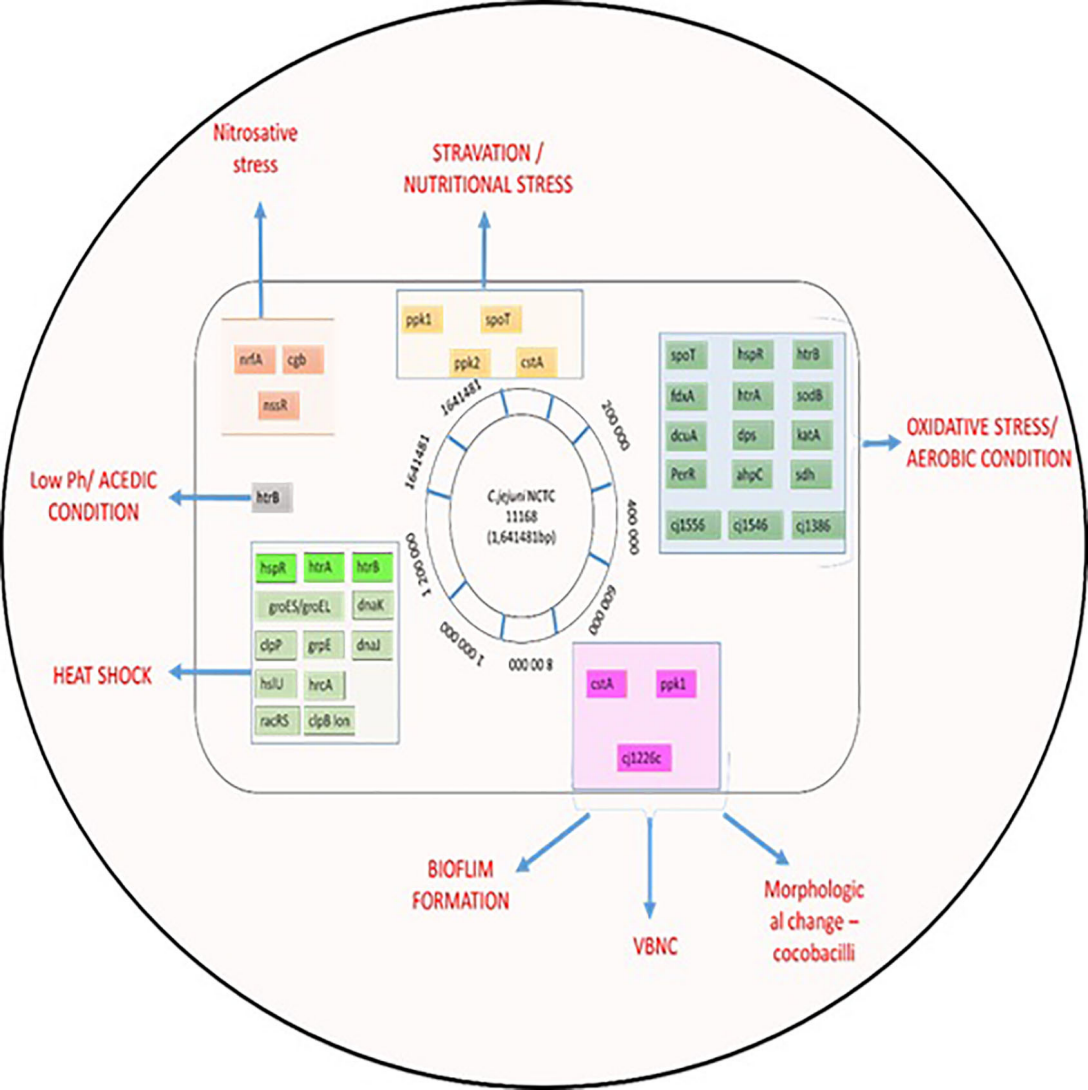
Summary of *C. jejuni* responses to stresses. The chromosome of *C. jejuni* NCTC11168 is represented by a black circle on which the location of genes, involved in different stress responses, are shown as colored lines. Genes are colored according to their role; gene names shaded in grey are involved in multiple stress responses.

The food matrix is one environmental factor that can influence micro-organism survival in the food system (all processes of production, processing, transport, and consumption) (de Oliveira et al., 2019; Farfán et al., 2019). After exposure to stress in the food system, expression of virulence and survival genes increased in *Listeria monocytogenes* (Olesen et al., 2009; Farfán et al., 2019). Day and Hammack (2019), reported enhanced gene expression under stress tolerance in *L. monocytogenes* in processed foods like meat and sausage juices compared with a laboratory setting. In contrast, stress-tolerance genes of *Lactobacillus sakei* were decreased in meat products (Prechtl et al., 2018), chicken meat and juice (Birk et al., 2004). Meat exudate, such as that from poultry meat, contains enzymes, myogens, myoglobin lactic acid, and amino acids (Wang et al., 2013). ‘Chicken juice’ can be used as a food-based model system for investigation of microbial survivability. Birk et al. (2004) recommended using the system to enhance understanding of *C. jejuni* viability on poultry products. C. jejuni survived longer in chicken juice (due to increased biofilm formation) stored at 5°C and 10°C (Brown et al., 2014). Ligowska et al. (2011) reported that expression of the gene *luxS* was increased in *C. jejuni* cultured in chilled poultry-meat juice. This highly conserved gene encodes the enzyme LuxS (S-ribosylhomocysteine lyase), which forms part of a quorum sensing system with autoinducer-2 (AI-2) and regulates gene expression. Differences in the recovery and identification of *Campylobacter* spp. between meat exudate and carcass rinse sampling methods in poultry have been demonstrated (Simmons et al., 2008; Duffy, 2019), as shown in **Figure 3**.

Previous research has shown that microbes form biofilms during food processing, such as in meat exudate conditions. Species of the genus *Salmonella* created a biofilm on the surface of stainless steel when cultured in laboratory media or meat exudate (Wang et al., 2013). Differences in the shape and cell density of mature biofilms were observed between food processing and laboratory environments. Longo and Spano (2019) reported the formation of biofilm in *L. monocytogenes* and species of the genera *Pseudomonas* and *Staphylococcus* on meat-treated surfaces, such as polyvinyl chloride, polyurethane, and steel. *C. jejuni* was more prone to forming biofilms in chicken juice than in a laboratory environment due to high nutrient availability (Brown et al., 2014). Thus, processed foods that contain many macronutrients are easily contaminated by microbes; these foods include the meat juice of chicken and beef, milk protein, and dairy products (Kusumaningrum et al., 2003; Healy et al., 2010).

## Viable but Non-Culturable (VBNC) State

Some microbes can endure unfavorable environments, such as nutrient deprivation, desiccation, inadequate pH, and temperature changes (Blanco-Lizarazo et al., 2018; Jin and Riedel–Kruse, 2018). Few microbes are capable of living in these unfavorable environments, but some organisms may enter a VBNC state for subsistence. Microbes in the VBNC state are unable to multiply, and their morphology is transformed into a coccoid shape (Poursina et al., 2018; Jin and Riedel-Kruse, 2018). Bacteria decrease their metabolism in the VBNC state but may retain the virulence capacity to infect a host and cause disease (Oliver, 2010; Fakruddin et al., 2013; Poursina et al., 2018). The VBNC state has been found in several micro-organisms, such as *C. jejuni*, *V. parahaemolyticus*, *Salmonella* ser. Typhi, and *Helicobacter pylori* (Azevedo et al., 2007; Zeng et al., 2013; Otigbu et al., 2018; Yoon and Lee, 2020). In an unfavorable environment, *C jejuni* can survive by using the VBNC tactic (Gangaiah et al., 2010; Zeng et al., 2013; Otigbu et al., 2018; Yoon and Lee, 2020). *C. jejuni* entered the VBNC state when cultured for 18–28 days at 4°C (Jones et al., 1991). Magajna and Schraft (2015) studied the VBNC status of planktonic cells and biofilm cells at 4°C and found that biofilm cells converted to VBNC status quicker than planktonic cells in nutritionally deprived and hostile-temperature environments. The VBNC form of *C. jejuni* affects CadF expression at 4°C (Otigbu et al., 2018). CadF protein is one of the elements influencing microbial invasion. The VBNC form of Campylobacter has been categorized based on reduced metabolism, augmented production of the degrading enzymes and substrates, and (Chaveerach et al., 2003; Upadhyay et al., 2019). Consequently, microbes can live for longer periods in hostile conditions (Kovacs et al., 2019).

## Adaptation to Major Environmental Stresses by *Campylobacter* spp.

Adaptation by *Campylobacter* spp. to various stresses such as acidic environment, salt tolerance, thermotolerance (heat and cold), UV stress, osmotolerance, desiccation, biofilm formation, and antibiotic resistance, are explained in detail in **Table 1**.

**Table 1:** Cluster of genes involved in the multiple stress responses of *C. jejuni*.

Sr. No	Target Mechanism	Gene	Stress tolerant Gene	Reference
1	Nitric Oxide and Nitrosative Stress in Campylobacter jejuni and Campylobacter coli	nrfA	Nitrite reductase, formate-dependent	Mühlig et al., 2014; Einsle (2011)
		cgb	Single-Domain Hemoglobin in Mediating Resistance to Nitric Oxide and Nitrosative Stress	Elvers et al., 2004; Pittman et al., 2007
		nssR	Single-Domain Hemoglobin in Mediating	Monk et al., 2008; Avila-Ramirez et al., 2013
2	Heat shock efficiency	htrB	Promotes Abiotic and Biotic Stress Tolerance in Transgenic Arabidopsis thaliana	Svensson et al., 2008; Poli et al., 2012
		htrA	high-temperature requirement A (HtrA)-like protease and chaperones in the cell envelope,	Svensson et al., 2008
		groES/groEL	Chaperonin	Laranjo and Oliveira, 2011
		dnaK	Chaperonin	
		clpP	Two promoters; roteolytic component of the Clp or Ti protease	Gerth et al., 1998
		grpE	Nucleotide sequence of a *Bacillus subtilis* gene homologous to the grpE gene	Völker et al., 1992
		dnaJ	Arabidopsis DnaJ (Hsp40) contributes to NaCl-stress tolerance	Zhichang et al., 2010
		hslU	Proteomics Analysis of Drought Stress-Responsive Proteins	Xu et al., 2009
		hrcA	Conserved ATP-dependent proteases of *C. jejuni* to stress tolerance and virulence	Chon et al., 2007
		racRS	Salinity stress tolerance - ascorbate-glutathione	Kang et al., 2013
		clpB lon	Protease ATP-dependent (*E. coli* ClpA) • ATPase activity	Parsell and Lindquist, 1993
3	Nutrition Depletion/Starvation	ppk1	Quorum sensing genes/inhibiting polyphosphate kinase	Sarabhai et al., 2015 Gangaiah et al., 2010
		spoT	cytosolic ascorbate peroxidase/peroxiredoxins	
		ppk2	The adenylate cyclase gene MaAC/membrane location of the protein	
		cstA	Arabidopsis genes	Auesukaree et al., 2009
4	Osmotic Tolerance	htrB	ATP binding cassette transporter components PaqP and PaqQ in bacterial salt stress tolerance	Lin et al., 2009a
		ppk1	Inhibiting polyphosphate kinase	Sarabhai et al., 2015
		cj1226c	Influences biofilm formation	Svensson et al., 2008; Svensson et al., 2009
5	Low pH/Acid Tolerance	htrB	ATP binding cassette transporter components PaqP and PaqQ in bacterial salt stress tolerance	Lin et al., 2009b
6	Oxidative Stress/Oxygen Stress	spoT	Quorum sensing genes/inhibiting polyphosphate kinase	Sarabhai et al., 2015
		hspS	Proteomics Analysis of Drought Stress-Responsive	Parsell and Lindquist, 1993
		htrA		
		fdxA	stress-responsive cyclophilin gene	Chen et al., 2007
		sodB	Resistance to peroxynitrite and stage-specific survival in macrophages	Master et al., 2002
		dcuA		
		dps		
		katA		
		perR		
		ahpC		
		sodB-sdh		
		cj1556	Additionally Influences biofilm formation	Svensson et al., 2009
		cj1546		
		cj1556-cj1386		

### Genes Involved in Stress Sensing/Adaptation

Acid-tolerance mechanisms: The adaptive tolerance response (ATR) was identified as the initiator of cross-protection for the survival of microbes under various stressful or unfavorable conditions (Oh et al., 2015), and was also found in foodborne pathogens (Li et al., 2018; Cariri et al., 2019; Mayton et al., 2019). Murphy et al. (2003) discovered an ATR in *C. jejuni* and a comparable result in the initiation of ATR was observed between stress-exposed and nonexposed organisms when the organism at the mid-exponential stage (8 h) was unable to start an ATR under air- and acidic-stress conditions. Conversely, stationary-phase (48 h) organisms could initiate ATR at pH 4.5 under air and acidic status compared to nonexposed organisms. They displayed acidic cross-protection, which initiated ATR under oxygen or air status. In addition, the ATR initiation of microbes at pH 4.5 varies according to the culture media; this might be due to the different nutrient compositions of the various culture media (Kovacs et al., 2019), -. C*. jejuni* demonstrated an ATR capacity at pH 4.5 when exposed to aerobic conditions with acidic and nutritional deprivation (Oh et al., 2017). Acidic stress initiated the upregulation of *perR* genes to counter oxidative disturbance.

Acid shock has a significant biological impact in situations of acidic pH and low (organic) acids. Fatty acids are carboxylic acids generated by fermentation, and include propionate, butyrate, and acetate (Luo et al., 2015; Eguchi and Utsumi, 2016). The fatty acids cause toxicity in their unloaded, protonated form because they may penetrate the plasma membrane, dissociate a proton, and create a lower intracellular pH.

An adaptive tolerance response to aerobic + acid conditions in *C. jejuni* (Oh et al., 2019) was shown to induce a global stress response mechanism (S.H Kim, unpublished data). An adaptive tolerance response (ATR) produced as a result of sub-chronic stress adaptive response and offers protection against subsequent lethal stress exposure (Noreen, 2019). We have defined an ATR in *C. jejuni* previously. The mediation of acid and oxygen concentration, makes them to adopt improved survival mechanism against lethal pH (Taylor et al., 2017). De novo protein synthesis was necessary for the initiation of ATR in *C.jejuni*, which implies enhanced protein synthesis occurred during the induction phase. During the induction of an ATR to acid stress, analysis of protein expression profiles demonstrated a global cellular response (S.H Kim, unpublished data). Based on MALDI-TOF mass spectrometry different Protein expressed during induction of the ATR in *C.jejuni*, which revealed that the majority of proteins were involved in modification, repair and biosynthesis.

The ATR in *C. jejuni* has been shown to incorporate up-regulation of generic stress proteins involved in protein defense or breakdown, such as the heat-shock response based on universal chaperones DnaK and GroEL, which are among the most highly conserved protein-coding genes known to be involved (Tang et al., 2017). Chaperone proteins may be involved in aerobic + acid denaturation or damage repair of proteins. Chaperone based GroEL and DnaK heat shock protein (HSPs) have been described as caused under acid conditions in *Salmonella typhimurium* (Ghazaei, 2017), which plays a major role after mild stress, either in the prevention of subsequent DNA damage or in the repair of already damaged DNA. The reported protein response were found to be closely associated with following pathogens such as *S. typhimurium* (Ghazaei, 2017), *Escherichia coli* (Burt et al., 2007) and *Acinetobacter baumannii* (Cardoso et al., 2010). This global reaction, in *C. jejuni*, which induced various mechanisms of survival and offers an initial insight into mechanisms that contribute to resistance of aerobic + acid susceptibility.

ATR-related RpoS: Transcription controller σs, encoded by the *rpoS* gene (RNA polymerase sigma factor), is a replacement sigma factor, the amount of which increases dramatically during any permanent stage of the microbes. The increase in σs concentration and gene expression is known to influence acid-shock proteins, such as high osmolality, low pH, hydration, and oxidation in cell survival (Ferreira et al., 2001). Sudden increases in cell acidification also cause strong increases in *rpoS* levels. Mutants that are defective in *rpoS* or that generate low concentrations of *rpoS* are highly susceptible to acidic conditions.

### Salt-Tolerance Mechanisms

Sodium chloride (NaCl) is one of the most used preservatives in the food industry. *C. jejuni* is highly responsive to high osmolarity compared to most other enteric microbes (Feng et al., 2018; Kovacs et al., 2019). *C. jejuni* is unable to multiply with ≥2% NaCl at 42°C, but can multiply in the presence of 0.5% to 1.5% NaCl at 42°C (Gomes et al., 2018). Lake et al. (2019) reported that *C. jejuni* could tolerate 7.5% sodium chloride (NaCl) in media at 4°C better than at 22–30°C as measured using bioluminescence. In microarray analysis, Zhao et al. (2019) found that *C. jejuni* had augmented expression of oxidative-stress genes and heat-shock genes after exposure to hyperosmotic conditions.

### Genetic Regulation by Sigma Factors

*C. jejuni* has a genome size of 1.4 Kbp, coding for approximately 1731 genes. In contrast to other environmental and food pathogens that have several gene-regulation processes occurring *via* sigma factors, *C. jejuni* has only three sigma factors (Wösten et al., 1998; Parkhill et al., 2000; Carrillo et al., 2004), and no recorded extracytoplasmic-function (ECF) sigma factors. The three sigma variables account for most operations related to gene regulation. Sigma 70 or RpoD is the housekeeping sigma factor that regulates most *C. jejuni* promoters. The other two sigma factors, sigma 28 (FilA, Filament A) and sigma 54 (RpoN), regulate 44 different genes that are mostly related to flagellar synthesis and protein secretion (Studholme and Dixon, 2003; Porcelli et al., 2013). The regulatory mechanisms and nucleic-base composition of the sigma-factor promoters were detailed by Petersen et al. (2003). Major promoters recognized by *C. jejuni* sigma subunits have the –10 element, whereas there is no consensus for the –35 element. The regulatory roles of RpoN in *C. jejuni* under various stress conditions were shown were shown using RpoN mutation and complementation in a study by Hwang et al. (2011). FilA is thought to regulate motility as well as the virulence of *C. jejuni* (Carrillo et al., 2004). Thorough genomic research into these mutant strains is required to elucidate the intricacies of gene regulation among the three sigma variables in this uncommon pathogen. Furthermore, how the lack of conservation of the –35 element contributes to optimal transcription *in vivo* remains to be determined. Morphological differences may exist, such as the conversion of a spiral bacterium to a coccus-/rod-shaped bacterium under osmotic and cold stress (Carrillo et al., 2004; Hwang et al., 2011). Even if *C. jejuni* is regarded as a pathogen transmitted *via* meat and poultry, it is not very tolerant to several nonoptimal conditions, particularly desiccation and osmotic stress.

### Role of Osmolytes in Cryotolerance

Compared with *Salmonella* spp. and *E. coli*-like enteric bacteria, little is known about the mechanisms that enable survival of *Campylobacter* spp. under various environmental and stress conditions. A previous study found that *C. jejuni’s* ability to influence gene expression after exposure to environmental stress was a barrier to comparison with other bacteria (Park, 2002). Rapid temperature decreases cause bacteria to express a distinct set of proteins, and this response is known as cold shock. These proteins are predominantly nucleases, helicases, and ribosome-related elements that communicate with and bind to RNA and DNA. Cold-shock proteins induce a membrane adaptation, cold signal sensing, and translation-device alteration (Ultee et al., 2019). Ultee et al. (2019) reported motility for oxygen consumption, protein synthesis, and *C. jejuni* survival capacity at 4°C. Lu et al. (2011) revealed that *C. jejuni* survive at in low-temperature. This indicates that *C. jejuni* may produce a cold-shock effect that influences low-temperature gene expression to 4°C. CspA is the main cold-shock protein in *C. jejuni*, and functions as an RNA chaperone to enhance more effective cold-shock protein translation (Parkhill et al., 2000; Giuliodori et al., 2010). It is not yet clear how *C. jejuni* respond to or regulate the expression of genes during cold shocks. Based on documented studies, the cold-shock reaction is presented as a complex system of genes that are regulated by the same stimulus, where post-transcriptional conditions are essential. *C. jejuni* poses problems to food security and public health in the food-processing industry, since it survives for several months at 4°C. *C. jejuni* declined by about 1 log cfu/ml when stored at 4°C for seven days (Guévremont et al., 2015; Lake et al., 2019). Oxidative stress can upregulate cold-shock protein expression, which can extend the life span of *C. jejuni* in hypothermal conditions (Karki et al., 2019).

### Survival During Ultraviolet (UV) Stress

VBNC refers to a state in which conventional culture on enhanced agar media does not detect microbial cells, although it remains feasible to resuscitate the microbes under preferential circumstances. This unique survival strategy has been shown to exist in nature (Salma et al., 2013). More than 60 different bacterial species have been found to be VBNC, including both Gram-negative (e.g., *E. coli*, *S. enterica*, *C. jejuni*, *H. pylori*, *Pseudomonas aeruginosa*, and species of the genera *Legionella* and *Vibrio*) and Gram-positive (e.g., species of the genus *Enterococcus*, *Micrococcus luteus*, and *L. monocytogenes*) species (Salma et al., 2013). Following a severe dose of UV (0.192 J/cm2), no viable *Campylobacter* cells were identified from the original level of 7 log cfu/ml in the liquid media (skimmed milk exposed to UV and diluted 1:4 with extreme rehabilitation diluents) (Xiong, 2009). Substantial variability of up to 4 log cfu/ml was observed in the susceptibility of *Campylobacter* isolates following UV treatment. In UV-treated (0.192 J/cm) fresh chicken fillet, *C. jejuni*, was decreased by 0.76 cfu/g, whereas, a reduction in *C. jejuni* of up to 3.97 log cfu/cm was attained with UV treatment of packaging and surface materials. These data indicated that *Campylobacter* is UV-prone, but concerning differentials occurred among the studied isolates. Overall, UV application could help improve the microbiological quality of raw chicken and remove contamination of related surfaces and packaging (Haughton et al., 2011).

Investigations were conducted concerning organism survival in rivers, coastal waters, and sewage to investigate the natural and artificial habitats of *C. jejuni* with UV-B light (280–315 nm) (Hénault-Ethier et al., 2016; García-Peña et al., 2017; Otigbu et al., 2018). Another research project in conjunction with these revealed that *C. jejuni* was susceptible to UV-C light (254 nm). UV sensitivity was greater than that of other microbes (Butler et al., 1987). The application of UV-C radiation to decrease *C. jejuni* in chicken breast also attracted interest (Rodrigues et al., 2019), as well as in broiler meat (Zhuang et al., 2019) and ready-to-eat ham (Yang et al., 2017). UV-light techniques have been extensively explored for reducing micro-organisms, including *Campylobacter*, in foodstuffs (Rodrigues et al., 2019; Zhuang et al., 2019).

UV irradiation achieved a maximal reduction of *C. jejuni* on broiler meat and broiler skin of 0.7 and 0.8 log, respectively. The maximal decrease by UV irradiation on broiler carcasses (254 nm, 32.9 m W/s per square inch) was 0.4 log, and the combination of UV and activated oxygen also achieved a 0.4 log reduction in *C. jejuni*. The primary sanitation method for *C. jejuni* in broiler carcasses cannot rely on UV irradiation alone or in conjunction with activated oxygen. However, application of these methods in conjunction with other sanitization techniques, as well as the adequate processing and sanitation of processing plants, may be more efficient than the use of these processes to reduce *C. jejuni* on broiler carcass surfaces (Isohanni and Lyhs, 2009). UV irradiation was less efficient at removing *C. jejuni* on broiler meat and skin than on agar plates. It reduces *C. jejuni* on grilled skin a little more effectively than on meat. Dry meat undergo ultraviolet radiation has low invasive capacity, and the cutting edges of food perhaps produced shade that interfered with UV irradiation (Rodrigues et al., 2019). The fibers could be isolated by swabbing the surfaces and allowing the swabs to absorb humidity from below the meat layer. After flaming, the skin did not appear to have changed much, and bacteria could not have crossed the threshold skin into the meat. Wong et al. (1998) also indicated that gram positive bacteria were more efficiently reduced by UV irradiation. However, the effects of UV irradiation can differ considerably in *C. jejuni* isolates from different origins and at different growth stages (Yaun et al., 2003).

### Oxidative Stress and Aerotolerance

*Campylobacter* does not usually grow in environments of atmospheric oxygen (air) due to it being microaerophilic and requiring 5–10% carbon dioxide (CO_2_) (Frirdich et al., 2019). *Campylobacter* can tolerate oxidative stress even after exposure towards aerobic conditions (Kim et al., 2015). Microaerophilic environment generates favorable growth conditions for *C. jejuni* (Geng et al., 2019). Karki et al. (2019) found that subcultures of *C. jejuni* could develop colonies on blood agar at 4, 37, and 42°C in air conditions. This exposure to aerobic conditions leads to the transformation of both the cell morphology and the pattern of the external membrane proteins. Their results indicated that the bacterial cells had high survivability in aerobic conditions compared to microaerobic conditions. Geng et al. (2019) reported that subcultures of *C. jejuni* from both sterile chicken mince and stream water developed colonies at 5, 25, and 37°C on blood agar, and that cells were more likely to survive when cultured in a microaerophilic than an aerobic environment.

In comparison with microaerobic conditions owing to oxidative pressure, *C. jejuni* showed external structural changes in the form of coccoid morphology (Oh et al., 2015), and the inner ATP synthesis of *C. jejuni* decreased with oxidative stress (Cain et al., 2019). Under microaerophilic environments, *C. jejuni* may develop better than under oxygenic conditions at a cell concentration of <10^5^ cfu/ml (Kaakoush et al., 2007).

### *C. jejuni* Heat-Shock Response

Heating is one of the sanitizing techniques used for food preservation in the food sector. Heat treatment readily reduces the survival of *C. jejuni* relative to other enteric micro-organisms. For *C. coli*, decimal reduction times (D-values) were 381, 89, 21.9, and 5.7 s at 49.9, 55.4, 60.0, and 62.5°C, respectively, in phosphate buffer saline (PBS) (Habib et al., 2010; Upadhyay et al., 2019). Treatment of *C. jejuni* at 55°C for 3 min, decreased the density by 2–3 log cfu/ml (Kovacs et al., 2019). Heat treatment caused *C. jejuni* to lose its invasion capacity, and upregulate transcriptional factor HrcA for acid shock (Xu et al., 2019).

### Desiccation Tolerance

Tolerance to desiccation in *Campylobacter* spp. was first reported by Fernandez et al. (1985) in several biotypes of *C. coli* and *C. jejuni* subjected to 2–8 hours of exposure. The RpoN sigma factor does not significantly contribute to the tolerance to osmotic shock or desiccation, whereas tolerance of cold or refrigeration temperatures can be directly correlated with bacterial survival capacity in cold environments (Burgess et al., 2016). The extreme sensitivity to desiccation and poor tolerance to heat and drying established that blowing hot air was an efficient method to prevent carrying dormant *C. jejuni* from poultry to human hosts in commercial settings (Berrang et al., 2011). Such methods could be applied to farms to prevent pathogenic carriers through poultry.

### Biofilm Formation and Stress Adaptation

**Figure 4:**
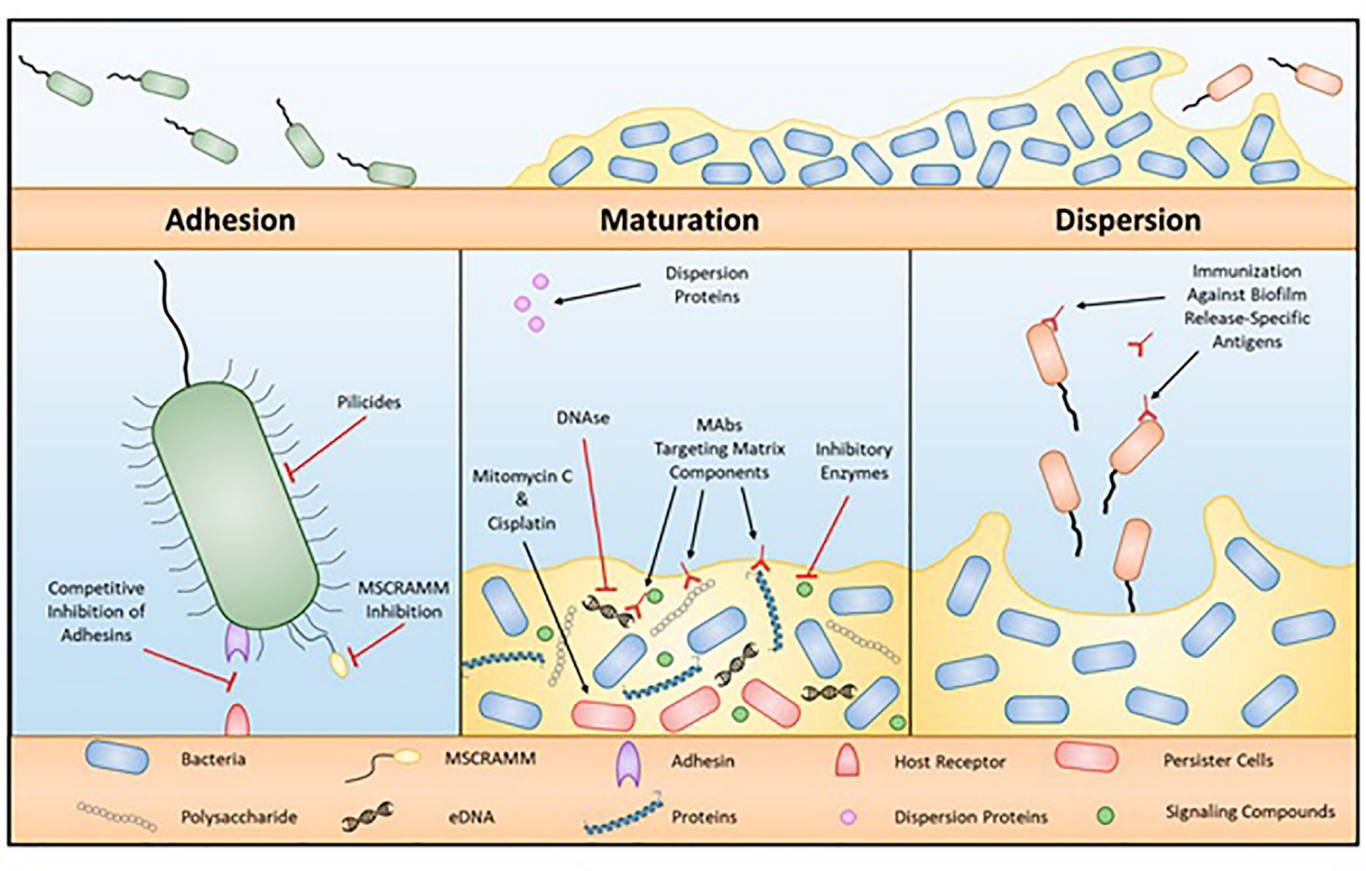
Process of biofilm formation.

Extracellular polysaccharide (EPS) accumulation leads to biofilm formation by microbes, biofilm formation could allow additional species to accumulate on surfaces (Simoes and Simões, 2013; Maes et al., 2019). EPSs compressed of nucleic acids, polysaccharides, proteins, phospholipids, and teichoic acids to form biofilms (Miao et al., 2019). Many factors stimulate biofilm formation, including temperature, NaCl, pH, compounds of food, and type of surface (Arnold and Silvers, 2000; Nguyen et al., 2006; Speranza et al., 2011; Vázquez-Sánchez et al., 2013; Mavri et al., 2016; Whitehouse et al., 2018; Longo and Spano, 2019; Xu et al., 2019). Biofilms can form on dairy-product-handling machinery and nutrition-handling surfaces (Miao et al., 2019), and can therefore contribute to the occurrence of foodborne diseases and create a public health issue (Maes et al., 2019; Miao et al., 2019). There are numerous reports on foodborne diseases in relation to biofilm development (Metselaar et al., 2015; Mavri et al., 2016; Whitehouse et al., 2018; Ma et al., 2019). Microbes in biofilms are more resistant to antibiotics than plankton cells are (Stewart and Costerton, 2001; Olsen, 2015). *C. jejuni* preconditions define their environment for growth, and Surface attachment and biofilm generation are vital tools for environmental stability (Dykes et al., 2003), as shown in **Figure 4**.

*C. jejuni* can generate biofilms in liquid media as a monospecies (Sałamaszyńska-Guz et al., 2018) in aerobic conditions (Ovesen et al., 2019) *C. jejuni* can form biofilms both as a monospecies and as a combination of microbes (The et al., 2019) and nutritional components (Bronnec et al., 2016). Sałamaszyńska-Guz et al. (2018) showed that the aggregating and pellicle form of *C. jejuni* that forms at 30–37°C in a microaerobic environment allows the bacteria to survive under aerobic conditions. Ovesen et al. (2019) demonstrated that *C. jejuni* easily creates biofilms, and that flagellar motility aggravated biofilm production. It currently reads as though it is the report of Ovesen et al., 2019 stated that *C. jejuni* could acclimate to develop a biofilm linked to CsrA under aerobic conditions (Askoura et al., 2016; Ye et al., 2019). Therefore, CsrA mutation leads to inhibition of biofilm formation (Fields and Thompson, 2008). *C. jejuni* can also contribute to biofilm formation in combination with other microbes under a microaerobic environment, but the combination is specific to the microbes and the environment (The et al., 2019), for example the poultry environment is an example of this specific environment/microbe combination. The biofilm formation capacity of *C. jejuni* depends on culture media, oxidative stress, temperature, and interspecies composition (Bronnec et al., 2016). Protein generation, quorum sensing, and flagellar sensing also influence the capacity of *C. jejuni* to generate biofilms, as shown in **Table 1**.

### Antibiotic Susceptibility of *C. jejuni*

Antibiotics are typically used to fight against bacterial infections (Pedersen et al., 2018), and possess different mechanisms to kill or inhibit bacteria. For example, quinolones, such as nalidixic acid, dysregulate DNA synthesis in microbial cells (Jacoby, 2005), whereas macrolides, including erythromycin, bind to ribosomes in the microbes, blocking elongation of the peptide loop (Arsic et al., 2018). Severe cases of campylobacteriosis require adequate treatment with antibiotics (Wieczorek and Osek, 2013), usually a fluoroquinolone and macrolide combination (Devi et al., 2019). Improper and frequent antibiotic use has led to increased antibiotic resistance in *Campylobacter*, which is a public health issue. Consequently, the fluoroquinolone and macrolide efficacy can fail to overcome the antibiotic resistance of *Campylobacter* (Pedersen et al., 2018; Bolinger et al., 2018; Silvan et al., 2018; Devi et al., 2019). The continuous usage of antibiotics such as tetracycline, ciprofloxacin, and erythromycin leads to the development of resistance in enteropathogens; specific resistance genes to these antibiotics were identified in *C. jejuni* isolates (Wirz et al., 2010), and comparable trends in *C. coli* were reported in Canada (Devi et al., 2019). Zwe et al. (2018) found that *C. jejuni* isolated from ducks in Singapore was resistant to ciprofloxacin (86.7%), nalidixic acid (84.4%), and erythromycin (11.1%) (Devi et al., 2019). The development of antibiotic resistance in *Campylobacter* means the treatment regime of campylobacteriosis will involve other antibiotics, like gentamycin (Aarestrup and Engberg, 2001; Pedersen et al., 2018).

## Conclusion

*Campylobacter* use a range of approaches for environmental and genomic survival, and molecular studies have facilitated a better understanding of these processes. Genetic modifications within the species *C. jejuni* have been significantly targeted, and genome sequencing for this species has been completed. Epidemiological studies and phenotypical analyses found variations in the incidence of strains of *C. jejuni*, or environmental circumstances between strains of *C. jejuni*. It has been easier to understand mechanisms that affect *C. jejuni* persistence by examining the transformation of this important pathogen in natural settings, such as soil and water, and combining connections with environmental changes. However, the reported differences in various strains of *C. jejuni* highlight the constraints of drawing generalized conclusions from individual strain research.

The multiple stress responses of *Campylobacter* spp. may facilitate survival in extreme environmental conditions, in addition to increasing resistance to subsequent traumatic conditions, which might enhance acquisition of virulence genes. Our review demonstrates the contribution of stress-tolerance responses to the resistance and pathogenicity of *C. jejuni*. Minor factors involved in stress management based on stress-responsible protein production are also involved in the activation and up- or down-regulation of virulence genes, and may contribute to the pathogenesis of *C. jejuni*. This finding is based on reported studies validated in different isolates of *C. jejuni* in response to stress adaptation, therefore caution should be taken in segregating and characterizing strains of *C. jejuni*. Gram-negative microaerophilic bacteria like *H. pylori* and *C. jejuni* are extremely common, and are human gastrointestinal pathogens. Only by combining these separate strands can the role of environmental survival in transmitting these important pathogens be fully understood.

### Required Future Research to Fill Current Knowledge Gaps

Major gaps in current research on stress responses on *C. jejuni*, so far, researchers have predominantly focused on antibiotic resistance and oxidative stress in *C. jejuni*. However, various other stress conditions and specific survival-mechanism-based evolutionary adaptation methods exist to overcome modern preservative conditions, such as acidity, alkalinity, osmotic imbalance, freezing, high temperatures, UV light, and dryness (reduced water content). Future research should concentrate on understanding the genetic make-up of *C. jejuni* that helps this organism survive various environmental conditions. Identification of these evolutionary adaptive mechanisms and specific signaling pathways will assist future researchers in developing effective methods to overcome the adaptive mechanism(s) of *C. jejuni*. Furthermore, understanding *C. jejuni* stress-oriented genes and their specific expression mechanisms based on environmental stressors have implications in biofilm interactions and their signaling mechanism(s), and in practical terms this could help with current technological hurdles in the food system.

## Author Contributions

The manuscript was written in detail and sectioned for specialized discussion with the respective authors in the field of research. Designing the outline of the review manuscript (Multiple stress tolerance in *Campylobacter jejuni*), visualization, and conceptualization—S-HK, RC, D-HO. Cross-protection and other general survival mechanisms towards environmental stress—SR. Genes involved in stress sensing/adaptation, acid tolerance mechanisms, protective mechanisms, systems for resistance to acid (AR1) or repressed by oxidants or glucose—AP. System of acid resistance 2 (AR2)/dependent on glutamate, system of acid resistance 3 (AR3)/arginine, ATR-reliant RpoS—EP, HJ, S-BH. Salt tolerance mechanisms—AP, RC. Genetic regulation by sigma factors—RC, S-HK, EB-M. Role of osmolytes in cryotolerance—RC, EB-M, FE, KB. *C. jejuni* heat-shock response—RC, S-HK. Desiccation tolerance—W-SB, AP. Biofilm formation and stress adaptation—RC, SR. Antibiotic susceptibility of *C. jejuni*—S-HK. All authors contributed to the article and approved the submitted version.

## Funding

Article processing charges have been covered by the Korea Research Fellowship Program through the National Research Foundation of Korea (NRF) funded by the Ministry of Science, in Young Researchers Program [2018007551].

## Conflict of Interest

The authors declare that the research was conducted in the absence of any commercial or financial relationships that could be construed as a potential conflict of interest.
